# Probucol, a “non-statin” cholesterol-lowering drug, ameliorates D-galactose induced cognitive deficits by alleviating oxidative stress via Keap1/Nrf2 signaling pathway in mice

**DOI:** 10.18632/aging.102337

**Published:** 2019-10-07

**Authors:** Jin-Lan Huang, Chao Yu, Min Su, Si-Man Yang, Fan Zhang, Yuan-Yuan Chen, Jin-Yuan Liu, Yi-Fan Jiang, Zhen-Guo Zhong, Deng-Pan Wu

**Affiliations:** 1 Jiangsu Key Laboratory of New Drug Research and Clinical Pharmacy, Pharmacy School, Xuzhou Medical University, Xuzhou, Jiangsu 221004, China; 2 Scientific research center of traditional Chinese medicine, Guangxi University of Chinese Medicine, Nanning, Guangxi 530200, China

**Keywords:** probucol, age-related neurodegenerative disease, oxidative stress, Keap1/Nrf2 pathway

## Abstract

Oxidative stress plays a vital role in the initiation and progression of age-related neurodegenerative diseases. Ameliorating oxidative damage is therefore considered as a beneficial strategy for the treatment of age-related neurodegenerative disorders. Probucol (Prob), a lipid-lowering prototype agent, was reported to treat cardiovascular diseases, chronic kidney disease and diabetes mellitus. However, whether Prob has an effect on age-related neurodegenerative diseases remains unknown. In the study, it was found that Prob ameliorated D-galactose (D-gal) induced cognitive deficits and neuronal loss in the hippocampal CA1 region. Moreover, Prob alleviated ROS and MDA levels by elevating SOD, GSH-PX and HO-1 mRNA and protein expressions, and improving plasmic and cerebral SOD and GSH-PX activities in D-gal treated mice. Furthermore, Prob promoted the dissociation of Keap1/Nrf2 complex leading to the accumulation of Nrf2 in nucleus, implying that the improved anti-oxidant property of Prob is mediated by Keap1/Nrf2 pathway. The study firstly demonstrates the favorable effects of Prob against cognitive impairments in a senescent mouse model, rendering this compound a promising agent for the treatment or prevention of age-related neurodegenerative disease.

## INTRODUCTION

Aging is a complicated multifactorial process causing a gradual decline in physiological function and the capability of an organism to survive. Oxidative damage has been increasingly considered as a contributing factor in the reduction of physiologic function occurring during the aging process [1]. Increasing evidence demonstrate that elevated oxidative stress in central nerve system (CNS) has been implicated to neuronal dysfunction in different kinds of age-related neurodegenerative disorders [2], such as Alzheimer’s disease (AD) [3] and Parkinson's disease (PD) [4]. It is well established that oxidative imbalance and resultant neuronal damage play vital roles in the initiation and progression of neurodegenerative diseases [5]. Thus, ameliorating oxidative damage is considered as a beneficial strategy for the treatment of age-related neurodegenerative disorders [6].

Oxidative damage in CNS may be ascribed to the failure of antioxidant defenses or the increased production of reactive oxygen species (ROS) [7]. The accumulation of ROS attack deoxyribonucleic acid, leading to neuronal damage and cognitive impairment [8]. It is well known that Keap1/Nrf2 signaling pathway plays a vital role in protecting cells from oxidative damage [9, 10]. Upon exposure to oxidative stress, Keap1 is incapable of ubiquitineating Nrf2, which allows Nrf2 to accumulate in the nucleus and activate its target genes, including antioxidant enzymes such as superoxide dismutase (SOD), glutathione peroxidase (GSH-PX) and Heme oxygenase-1 (HO-1), which in turn, scavenges cellular oxidative stress [9]. Increasing evidence has shown that Keap1/Nrf2 signaling is associated with the pathology of neurodegenerative disorders [11]. It has been reported that disturbed activity and expression of Nrf2 were observed in the patients and mouse model of AD and PD [12–14]. Pharmacological activation of Nrf2 or elevation of Nrf2 expression by lentiviral ameliorates the phenotypes of AD and PD [12], implying that Keap1/Nrf2 signaling may serve as a potential target against age-related CNS disorders.

Probucol (Prob) is a phenolic lipid-lowering prototype agent clinically used to treat cardiovascular diseases [15, 16]. In experimental and clinical studies, Prob has been reported to treat chronic kidney disease and diabetes mellitus due to its antioxidant and anti-inflammatory properties [17, 18]. In CNS, Prob possesses the capacity to attenuate oxidative damage by increasing GSH-PX activity, thereby playing protective roles in experimental models of neurotoxicity/neuropathology [19, 20]. Moreover, Prob could prevent cognitive and hippocampal synaptic impairments in amyloid β peptide- and STZ-injected mice [21, 22]. However, it is unclear whether Prob has a neuroprotective effect in age-related disorders.

D-galactose (D-gal)-induced aging model in rodents has been extensively considered as an animal model for brain aging in a variety of anti-aging studies since D-gal, which induces oxidative injury in CNS, resulting in memory and synaptic dysfunction [23], mimics the characteristics of the natural process of brain aging [24]. Therefore, in the present study, D-gal-induced aging model mice were intraperitoneally administrated Prob for 6 consecutive weeks to characterize the effect of Prob on cognitive deficit and neuronal damage induced by oxidative stress in aging model mice, thereby providing a potential opportunity for research regarding the pharmaceutical prevention and treatment of age-related disorders.

## RESULTS

### Effect of Prob on plasma cholesterol, organ index and general appearance in mice

**Figure 1:**
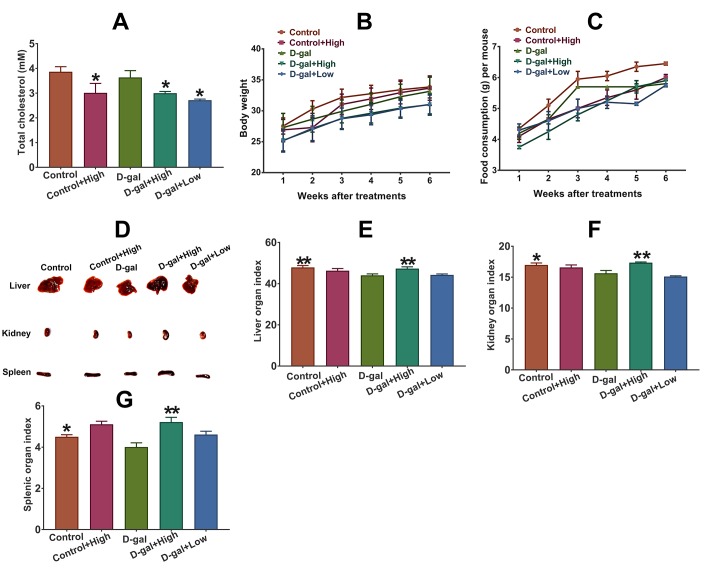
**Effect of Prob on plasma cholesterol, organ index and general appearance in mice.** (**A**) Effect of Prob on plasma cholesterol level. (**B**, **C**) Effect of Prob on body weight and food consumption of mice, respectively. (**D**) The representative picture of liver, kidney and spleen of mice. (**E**–**G**) Effect of Prob on liver, kidney and splenic organ index, respectively. Data in each experiment represent mean ± SEM from 10 independent samples. Statistically significant differences were calculated by one-way ANOVA using the SPSS 20.0 software. *P<0.05 and ** P<0.01, versus D-gal group.

As shown in Figure 1A, the levels of total cholesterol (TC) in Prob-treated groups significantly decreased compared to Prob-untreated groups, indicating the hypocholesterolemic effect of Prob. The body weight and food consumption of each mouse were also observed during Prob treatment. The results showed that Prob treatment did not affect body weight and food consumption of mice, suggesting that Prob treatment did not affect the general appearance of mice (Figure 1B and 1C). Additionally, the organ indices of liver, spleen and kidney in D-gal group were significantly reduced compared to the control group, whereas, Prob treatment markedly reversed D-gal-induced decrease in organ indices, implying that Prob may prevent D-gal-triggered organ atrophy (Figure 1D–1F).

### Prob treatment improves the cognitive deficits in D-gal-induced aging model mice

### MWM test

**Figure 2:**
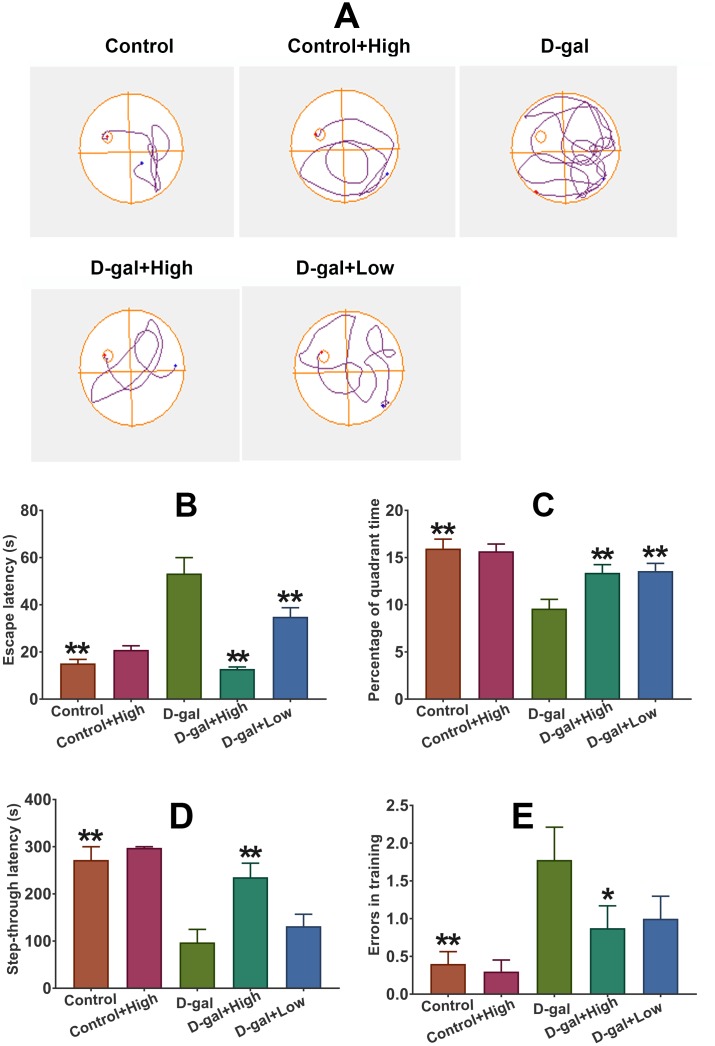
**Prob treatment improves the cognitive deficits in D-gal-induced aging model mice.** (**A**) The representative traces during the probe test. (**B**, **C**) Effect of Prob on escape latency and percentage of quadrant time in the MWM test, respectively. (**D**, **E**) Effect of Prob on step-through latency and errors in training in passive avoidance test. Data in each experiment represent mean ± SEM from 10 independent samples. Statistically significant differences were calculated by one-way ANOVA using the SPSS 20.0 software. *P<0.05 and ** P<0.01, versus D-gal group.

The spatial learning and memory was detected using MWM test. After four-day training, escape latency and percentage of time in the target quadrant on day 5 were recorded. As illustrated in Figure 2A and 2B, escape latency in D-gal was markedly longer than that in the control group, while the percentage of quadrant time significantly increased in the control group than that in the D-gal group, indicating the impaired cognitive function was induced by D-gal. Notably, Prob could remarkably shortened escape latency and prolong the quadrant time in the targeted quadrant of mice treated with D-gal (Figure 2A and 2B), implying that Prob holds the potential to improve learning and memory deficits induced by D-gal.

### Passive avoidance test

The retention memory was evaluated using the 24h retention trial of the passive avoidance test. The results showed that higher memory errors and poorer retention latency in D-gal group than that in the control group was observed (Figure 2A and 2B), suggesting that D-gal induces retention memory impairments. Importantly, Prob treatment significantly reversed D-gal-induced increased memory errors and declined retention latency (Figure 2A and 2B), implying that Prob possess the capacity to ameliorate D-gal-triggered retention memory loss.

### Prob treatment alleviates hippocampal neuronal loss in D-gal-induced aging mice

**Figure 3:**
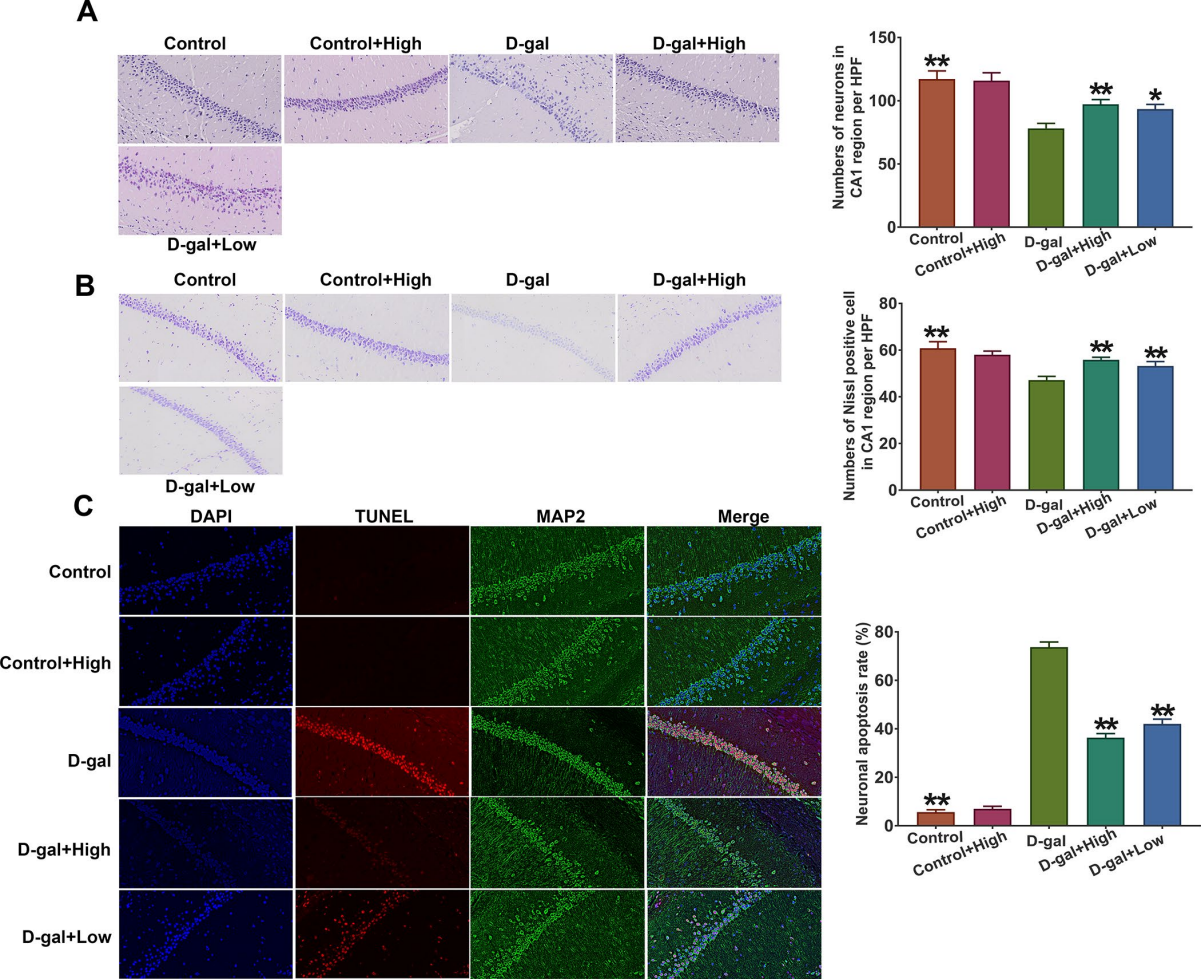
**Prob treatment alleviates hippocampal neuronal loss in D-gal-induced aging mice.** (**A**) H&E staining. (**B**) Nissl staining. (**C**) Cell apoptosis was detected by the TUNEL assay. The representative sections of hippocampal CA1 region were chosen and used to quantify positive cell numbers by a blinded observer. Data in each experiment represent mean ± SEM from 4-5 independent samples. Statistically significant differences were calculated by one-way ANOVA using the SPSS 20.0 software. *P<0.05 and ** *P*<0.01, versus D-gal group.

It has been documented that neurons in hippocampal CA1 region are highly susceptible to oxidative stress, which may cause neuronal loss and morphological change. Thus, in the present study, the hippocampal neurons in the CA1 region were chosen to be stained by H&E and morphology of neurons in the CA1 region was observed using 200×light microscopy. The results showed that the hippocampal neurons in the control group were arranged in an organized pattern with clear boundaries, whereas the hippocampal neurons of D-gal group exhibited loose and irregular arrangements and condensed nucleus was observed (Figure 3A). Importantly, the neurons in hippocampal CA1 region of Prob group displayed regularly and densely and fewer karyopyknotic neurons were found (Figure 3A). Additionally, using Imaging-Pro-Plus software, the number of neurons in hippocampal CA1 region was calculated. The results that the number of neurons in D-gal group was significantly lower than control group, whereas, Prob treatment markedly increased neuronal numbers as compared to D-gal group (Figure 3A).

Neuronal numbers in the CA1 region were also evaluated using Nissl staining. The results revealed that the number of Nissl positive neurons in D-gal group was remarkably decreased compared with the control group, while Prob treatment inhibited the reduction of neuronal numbers induced by D-gal (Figure 3B), indicating that Prob could rescue neuronal loss in the CA1 region. Additionally, cell apoptosis of CA1 region in D-gal group was higher than that in the control group, while Prob treatment restrained D-gal-induced apoptosis (Figure 3C), suggesting the inhibitory role of Prob in neuronal apoptosis.

Collectively, these results suggest that Prob treatment has an ability to alleviate hippocampal neuronal loss in D-gal-induced aging mice.

### Prob treatment ameliorates D-gal-induced oxidative stress in D-gal-induced aging mice

**Figure 4:**
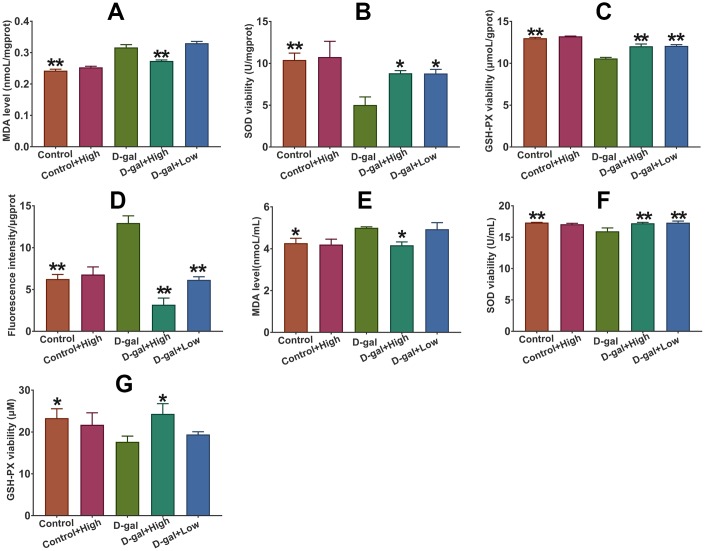
**Effect of Prob on ROS and MDA level, and the activities of SOD and GSH-PX.** (**A**–**C**) MDA level and the activities of SOD and GSH-PX in plasma, respectively. (**D**–**G**) Level of ROS and MDA, and the activities of SOD and GSH-PX in plasma, respectively. Data in each experiment represent mean ± SEM from 10 independent samples. Statistically significant differences were calculated by one-way ANOVA using the SPSS 20.0 software. *P<0.05 and ** *P*<0.01, versus D-gal group.

As shown in Figure 4, the cerebral ROS and MDA levels in D-gal group were significantly higher than those in the control group, indicating that D-gal triggers oxidative stress in the brains of aging mice. Notably, Prob treatment remarkably inhibited the increased levels of ROS and MDA in D-gal-treated mice (Figure 4A, 4D and 4E), suggesting that Prob can ameliorate D-gal-induced cerebral oxidative stress. However, Prob did not affect MDA and ROS levels in normal mice.

### Prob improves the activity of SOD and GSH-PX in D-gal-induced aging mice

The amelioration of oxidative stress by Prob in D-gal-induced aging mice suggests that Prob may influence the activities of antioxidant enzymes. The results showed that the activities of SOD and GSH-PX in the plasma and brains of D-gal group was significantly reduced compared to control group (Figure 4B, 4C, 4F and 4G). Importantly, Prob treatment improved the activities of SOD and GSH-PX in the plasma and brains of D-gal-induced aging mice (Figure 4B, 4C, 4F and 4G). Nevertheless, Prob did not impact on the activities of SOD and GSH-PX in the plasma and brains of normal mice (Figure 4B, 4C, 4F and 4G).

### Effect of Prob on the expression of SOD, GSH-PX and HO-1 in D-gal-induced aging mice

**Figure 5:**
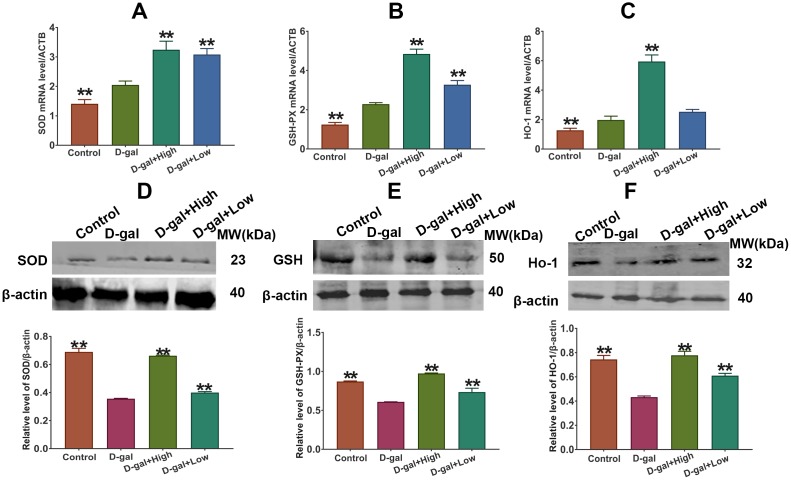
**Effect of Prob on SOD, GSH-PX and HO-1 expression.** (**A**–**C**) SOD, GSH-PX and HO-1 mRNA expression, respectively. (**D**–**F**) SOD, GSH-PX and HO-1 protein expression, respectively. Data in each experiment represent mean ± SEM from 10 independent samples. Statistically significant differences were calculated by one-way ANOVA using the SPSS 20.0 software. ** *P*<0.01, versus D-gal group.

As illustrated in Figure 5, the mRNA levels of cerebral SOD, GSH-PX and HO-1 in D-gal group were markedly higher than those in the control group. However, the protein levels and activities of SOD, GSH-PX and HO-1 in the brain were remarkably declined in D-gal group as compared to control group (Figure 5). Importantly, Prob treatment elevated mRNA and protein levels of cerebral SOD, GSH-PX and Ho-1 of D-gal-induced aging mice (Figure 5).

### Effect of Prob on the expression of Keap1/Nrf2 pathway

**Figure 6:**
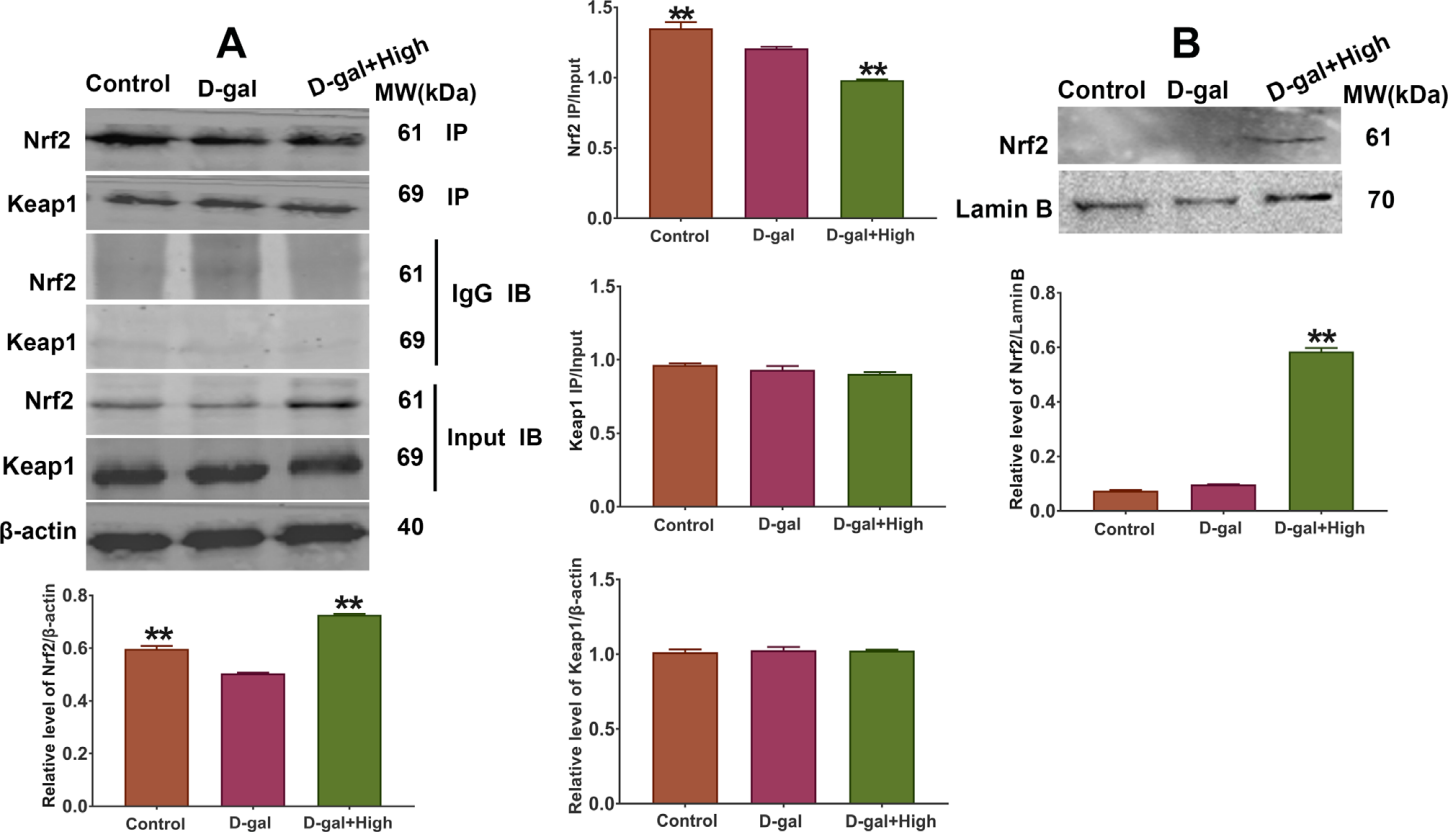
**Effect of Prob on the expression and dissociation of Keap1/Nrf2 complex.** (**A**) The dissociation of Keap1/Nrf2 complex was assessed by Co-IP assay. (**B**) The protein expression of Nrf2 in nucleus was assessed by Western blot assay. Data in each experiment represent mean ± SEM from 4-5 independent samples. Statistically significant differences were calculated by one-way ANOVA using the SPSS 20.0 software. ** *P*<0.01, versus D-gal group.

Since the expression of *SOD, GSH-PX* and *Ho-1* gene is regulated by Keap1/Nrf2 pathway, we speculated that Prob might have impacts on Keap1/Nrf2 signaling expression. The results of Western blot assay showed that the levels of Keap1 and Nrf2 in D-gal group did not exhibit significant difference compared to control group (Figure 6). Notably, Prob could enhance Nrf2 expression in the brains of D-gal-induced aging mice (Figure 6). However, Prob had no effect on cerebral Keap1 expression (Figure 6). These results indicate that Keap1/Nrf2 pathway is involved in the enhanced expression of antioxidant enzymes by Prob.

### Prob promotes dissociation of Keap1/Nrf2 complex leading to the accumulation of Nrf2 in nucleus

It has been well established that oxidative stress leads to the dissociation of Keap1/Nrf2 complex promoting Nrf2 to accumulate in the nucleus and activate gene expression of antioxidant enzymes, which in turn, ameliorates cellular oxidative stress. Thus, we investigated whether Prob has an impact on the dissociation of Keap1/Nrf2 complex. The results of Co-IP analysis showed that the ratio of ip/input of Nrf2 in D-gal group remarkably reduced as compared to control group (Figure 6A), indicating that D-gal promotes the uncoupling of Keap1/Nrf2 complex. Importantly, Prob treatment facilitated the dissociation of Keap1/Nrf2 complex as represented by a significant decrease in the ratio of ip/input of Prob group than D-gal group (Figure 6A). Additionally, the nuclear expression of Nrf2 in Prob group was markedly increased than that in D-gal group (Figure 6B), implying that Prob promotes dissociation of Keap1/Nrf2 complex leading to the accumulation of Nrf2 in nucleus.

## DISCUSSION

Age-related neurodegenerative disorders, characterized by progressive loss of neurons, affect millions of people every year. Unfortunately, there is no effective treatment for the disorders so far. It has been well established that elevated oxidative stress may trigger cellular damage, the deficit of the DNA repair system and impairment of mitochondrial function, thereby leading to an acceleration of the aging process and neurodegenerative diseases [25]. Thus, exploring potential candidates against oxidative stress would be considered as critical and efficient therapy for the treatment of age-related neurodegenerative diseases. The current study reveals that Prob, a phenolic lipid-lowering agent, holds the potential to ameliorate cognitive impairment and neuronal loss in D-gal-induced aging mice by attenuating oxidative stress via Keap1/Nrf2-mediated pathway (Figures 2–4). To the best of our knowledge, this is the first *in vivo* study reporting the profitable effects of this “non-statin” cholesterol-lowering agent on age-related neurodegenerative disease.

It has been confirmed that the aging process is characterized by changes in appearance, including significant decrease in organic atrophy caused by oxidative stress-induced apoptosis, which can be accelerated by the administration of D-gal [26]. In the present study, we found that the organ indices of liver, spleen and kidney in D-gal group were significantly reduced compared to control group, indicating chronic D-gal administration causes organ atrophy. Notably, Prob treatment significantly attenuate the decline in organ indices induced by D-gal, suggesting Prob has a prominent anti-aging effect in D-gal-induced aging model.

Chronically administrated with D-gal induces an accumulation of galactitol, leading to ROS generation via inhibiting Nrf2 activity [27]. Excessive ROS production causes cellular injuries by attacking DNA, proteins and lipids, resulting in the impairment of cellular function and accelerated aging [28]. In CNS, D-gal triggers neuronal degradation and cognitive dysfunction by inducing oxidative stress [24]. Thus D-gal induced aging animal has been considered as a rodent model for the research of age-related diseases [24, 27]. In the present study, D-gal administration led to an remarkable increase in oxidative stress as shown by higher levels of cerebral ROS and MDA than control mice (Figures 4 and 5). Additionally, D-gal caused neuronal loss in hippocampal CA1 region and cognitive impairment of mice (Figures 4 and 5), implying a senescence model was successively induced by D-gal.

Patients with age-related neurodegenerative diseases manifest progressive decline in cognitive function [29]. Patients present short-term memory loss in early stage and manifest long-term memory deficit in advanced stage [30]. In the study, MWM and passive avoidance test were performed to assess the role of Prob in D-gal induced impairment of cognitive function. The results of MWM test showed that Prob remarkably shortened escape latency and significantly elevated the percentage of quadrant time of mice treated with D-gal (Figure 2A). Additionally, the results of the passive avoidance test revealed that Prob significantly reduced memory errors and prolonged (Figure 2B). These results indicate that Prob holds the potential to ameliorate D-gal-induced cognitive impairment.

Oxidative imbalance and subsequent neuronal injury may induce neuronal degeneration and death, causing cognitive impairment in age-related neurodegenerative disease [28]. Antioxidant enzymes including SOD, GSH-PX and HO-1 are responsible for scavenging cellular oxidative stress [8]. In the study, we found that the mRNA levels of these enzymes were significantly increased in D-gal group relative to the control group, whereas the levels of enzymatic proteins were remarkably decreased in D-gal-treated mice as compared to the control mice (Figures 4 and 5), indicating that D-gal may affect protein degradation of the enzymes. Importantly, the results showed that Prob had the capability to eliminate ROS and MDA production by improving the expressions and activities of SOD and GSH-PX, and the expression of HO-1 (Figures 4 and 5), demonstrating an antioxidant capacity of Prob.

Keap1/Nrf2 signaling has been considered as one of the important antioxidant pathways preventing cells from oxidative stress [11, 12]. We speculated that the antioxidant capacity of Prob may be through Keap1/Nrf2 pathway. Thus, the effect of Prob on the expression of Keap1/Nrf2 pathway was investigated in the study. The results showed that Prob could remarkably improve Nrf2 expression, but not Keap1 expression (Figure 6), which is consistent with a previous study showing that Prob could activate Nrf2/ARE pathway by elevation Nrf2 expression, thereby inhibiting inflammation and neuronal apoptosis after spinal cord injury [31]. It has been well documented that the dissociation of Keap1/Nrf2 complex caused by oxidative stress facilitates Nrf2 to locate in the nucleus, leading to the activation of its downstream antioxidant genes’ expression [9]. We then investigated whether Prob has effects on the dissociation of Keap1/Nrf2 complex. The results revealed that Prob treatment promoted the dissociation of Keap1/Nrf2 complex and the accumulation of Nrf2 in the nucleus (Figure 6), indicating that Keap1/Nrf2 pathway is involved in the improved anti-oxidant property of Prob. Nevertheless, the mechanisms underlying the dissociation of Keap1/Nrf2 complex by Prob need to be investigated in further study.

Nrf2 activation has been reported to ameliorate AD and PD phenotypes [12]. Thus, pharmacological activation of Nrf2 has been considered as a benefit strategy to treat AD and PD [13, 32]. The present results, showing that activation of Nrf2 by Prob improves D-gal-induced neuronal loss and cognitive impairments via attenuating oxidative stress, provide support for the hypothesis that Prob may be a potential agent to treat AD and PD.

It has been reported that high cholesterol level contributes to the increases in markers of oxidative stress, thereby being associated with the development of neurodegenerative disease [33, 34]. It is likely that the hypocholesterolemic effect of Prob may contribute to its beneficial effects against neurodegenerative disease by modulating oxidative stress. In order to investigate the possibility, the effects of Prob on oxidative stress were investigated after D-gal-untreated mice were administrated with high-dosage Prob for six weeks. The results showed that Prob treatment could significantly reduce cholesterol level, but did not affect the levels of ROS and MDA, and the activities of antioxidant enzymes compared to the control mice (Figures 4 and 5), indicating that under physiological conditions, the cholesterol-lowering effect of Prob does not affect the state of oxidative stress. It should be noted, however, that after mice were treated with D-gal, Prob treatment could not only decrease cholesterol level, but also attenuate oxidative stress as shown by improving the expressions and activities of antioxidant enzymes and reducing ROS and MDA levels (Figures 4 and 5), suggesting that the antioxidant effect of Prob may be associated with the reduction of Prob in cholesterol level in pathological event.

Some limitations have to be interpreted. Firstly, more animal models of neurodegenerative disease, such as AD and PD animal models, should be used to investigate whether Prob has influences on AD and PD. Secondly, since high cholesterol level was reported to contribute to the increase in oxidative stress [33, 34], it cannot be ruled out the possibility that the hypocholesterolemic effect of Prob may alleviate oxidative stress. Further studies should be designed to explore whether the cholesterol-lowing effect of Prob is involved.

Taken together, the current study presents that Prob has the potential to ameliorate D-galactose induced cognitive deficits by alleviating oxidative stress via Keap1/Nrf2 signaling pathway. The study firstly reports the beneficial effects of Prob against D-gal-induced cognitive deficits and neuronal loss, rendering this compound a promising agent for further studies on the search for therapeutic strategies to treat or prevent age-related neurodegenerative disease.

## MATERIALS AND METHODS

### Drugs and reagents

Prob was obtained from Shanghai Aladdin Bio-chem Technology Co., Ltd (Shanghai, China). SOD, GSH-PX activity kits, MDA and ROS detection kits were purchased from Nanjing Jiancheng Bioengineering Institute (Nanjing, China). Keap1, Nrf2, SOD and GSH-PX antibodies were from Santa Cruz Biotechnology, Inc. (Texas, USA). Ho-1 antibody was from Merck Millipore (Massachusetts, USA). Other reagents were all provided from Sigma-Aldrich unless otherwise noted.

### Animals, drug treatment, and tissue preparation

Six- to eight-week-old adult male Kunming strain mice obtained from Xuzhou Medical University were randomly divided into five groups: Control group, Control+ Prob high-dosage group, D-gal group, D-gal+ Prob high-dosage group and D-gal+ Prob low-dosage group. The low- and high-dosage Prob-treated groups were intraperitoneally injected with 10 and 20mg/kg Prob daily, respectively; D-gal-treated groups were given subcutaneously with D-gal (150mg/kg) for six weeks. The treatment regimens of Prob and D-gal were according to previous studies [22, 35]. Control and D-gal groups were administrated intraperitoneally with the same volume of distilled water. The mice were euthanatized while the brains were excised for H&E staining, activity detection of antioxidant enzymes, western blotting, and real-time PCR assay. Moreover, liver, kidney and spleen of mice were excised for calculating liver, kidney and splenic organ index respectively. The organ index was calculated using the following equation: organ index = organ weight (g)/ body weight (g) × 100%. Animal care and experimental procedures were implemented in accordance with the document “Guidance Suggestions for Caring for Laboratory Animals” produced by the Ministry of Science and Technology of China in 2006.

### Morris water maze test

Cognitive function was detected by Morris Water Maze (MWM) test as previously described [36, 37]. The mice were subjected to four days of training trials. In each trial, the mice were trained to locate the hidden platform. On day 5, the capacity of spatial memory was detected using a spatial probe test. In the test, the percentage of time spent in the quadrant where the former platform was placed was recorded.

### Passive avoidance test

Passive avoidance test was performed for measuring memory retention in mice according to a previous study [38]. Briefly, the mice were placed individually in the illuminated compartment of automated passive avoidance system. By pressing the pedal, the apparatus was activated. The guillotine door was raised after 10 seconds allowing the mice to enter the dark compartment. The door was then closed and mice were given a foot-shock (0.2mA), for 2 seconds on training day. 24h after the training session, memory errors and step-through latency were recorded. If mice did not enter the dark compartment within 5min, the mice were assigned a score of 300 seconds.

### Haematoxylin and eosin (H&E) staining

The H&E staining assay was performed according to previous studies [39, 40]. Briefly, after brains were excised and fixed with formaldehyde for 24h, samples were rehydrated and embedded in paraffin. Sections of brain were prepared and stained by haematoxylin and eosin. The histological changes were observed by a light microscope (Olympus BX-50, Japan).

### Nissl staining

Nissl staining was performed according to the manufacturer’s instructions (Beyotime Biotechnology, China). In brief, ethanol-dehydrated samples were prepared and stained with Nissl staining solution for 10min at 37 °C. The total number of cells at CA1 region in each section was counted.

### TUNEL staining

TUNEL staining assay was used to determine the degree of cell apoptosis in hippocampal CA1 region according to the manufacturer’s instructions (Meilun Biotechnology, China). Sections were also stained for neuronal marker microtubule-associated protein (MAP2) (1:200, Proteintech, USA), with nuclei stained with DAPI, to identify neurons in hippocampal CA1 region. The percentages of neurons with apoptotic nuclei (both MAP2 and TUNEL positive) were then quantified.

### Real-time PCR

RT-PCR assay was performed as previously described [41, 42]. After extraction of mRNA and reverse transcription, samples were mixed with SYBR Green Master Mix and primers, and then, the reactions were performed. The primer sequences were as follows: SOD (105bp), sense (5′- GGCAAAGGTGGAAATGAAGA-3′), antisense (5′- CTCAGACCACACAGGGAATG-3′); GSH (184bp), sense (5′- CGAGCGAGTGGTGACGTA TG-3′), antisense (5′- ACGGGCAGTATAGTCGTCCT-3′); HO-1 (117bp), sense (5′- GAACCCAGTCTATGCC CCAC -3′), antisense (5′- GGCGTGCAAGGGATGATT TC -3′); surviving (105bp), sense (5′-CGTCACTTTGT CTGCCACTC-3′), antisense (5′-TCAGCCCTTAGCAC TCACCT-3′); ACTB (130bp), sense (5′-GTGCTATGT TGCTCTAGACTTCG-3′), antisense (5′- ATGCCACAG GATTCCATACC-3′).

### Western blot assay

Western blot assay was conducted as previously described [37, 43]. The protein samples were extracted and separated by SDS-polyacrylamide gel electrophoresis in 10% Tris-glycine gels and transferred to a nitrocellulose membrane. Primary antibodies including Keap1 (dilution: 1:500), Nrf2(dilution: 1:500), SOD (dilution: 1:500), GSH-PX (dilution: 1:500) and Ho-1 (dilution: 1:500) were incubated at 4 °C overnight, followed by incubation with IRDye purified secondary antibody (dilution: 1:10000). The immunopositive bands were visualized at Ex/Em=778 nm/795 nm.

### Cholesterol level detection

Total cholesterol level was measured in plasma using an enzymatic method according to the oxidase/peroxidase system using commercial kit reagents (Nanjing Jiancheng Bioengineering Institute, Nanjing, China).

### SOD, GSH-PX activity and MDA detection

SOD, GSH-PX activity and MDA level were detected according to SOD, GSH activity and MDA detection assay kit instructions, respectively. In brief, action buffers were added to the supernatants of tissue samples. The mixtures were incubated at 37 °C and the optical densities were measured at 550nm, 412nm, and 532nm using visible spectrophotometer for SOD, GSH-PX activities and MDA, respectively. The activities of SOD and GSH-PX were expressed as the μmoL per g of protein sample, and the level of MDA was expressed as nmoL/mL.

### ROS detection

ROS level was detected according to ROS detection assay kit instruction. Briefly, 100nM DCFH-DA was added to single-cell suspension for 30min. Then cells were collected, washed and detected at 485nm excitation wavelength and 525nm emission wavelength. The fluorescence intensity of ROS was expressed as fluorescence intensity per μg protein.

### Co-Immunoprecipitation (Co-IP) assay

Co-IP assay was performed according to a previous study [44]. Briefly, brain samples were lysed in iced cold PIPA buffer and the supernatants were collected by centrifuging at 14000rpm. The lysates were incubated with Keap1 antibody and protein A/G agarose gel overnight. The gel was washed with RIPA buffer, boiled with SDS loading buffer and subjected to SDS-PAGE resolution and Western blot assay. Nrf2 antibody was used to detect the interaction of these two proteins.

### Quantification analysis

For H&E, Nissl and TUNEL/MAP double staining assays, cells were analyzed according to a previous study [45]. Briefly, the sections approximately at the center of the hippocampal CA1 region were selected at 100 μm intervals from 3.2 mm anterior to the bregma to 6.8 mm posterior to the bregma, and the neuronal numbers of 5 sections of each sample per group were analyzed by a blinded observer using Imaging-Pro-Plus 6.0 software (Media Cybernetics Inc., America).

### Statistical analysis

The data of each group were presented as mean±SEM. One-way analysis of variance (ANOVA) was used to analyze the variance using SPSS software for Windows 20.0. P<0.05 was considered to be statistically significant.
